# Breast cancer in women with previous gestational diabetes: a nationwide register-based cohort study

**DOI:** 10.1186/s13058-024-01908-4

**Published:** 2024-11-04

**Authors:** Maria Hornstrup Christensen, Christina Anne Vinter, Thomas Bastholm Olesen, Maria Houborg Petersen, Ellen Aagaard Nohr, Katrine Hass Rubin, Marianne Skovsager Andersen, Dorte Moeller Jensen

**Affiliations:** 1 Steno Diabetes Center Odense, Odense University Hospital, Odense, Denmark; 2 Department of Gynecology and Obstetrics, Odense University Hospital, Odense, Denmark; 3 Department of Clinical Research, University of Southern Denmark, Odense, Denmark; 4 Research unit OPEN, Department of Clinical Research, University of Southern Denmark, Odense, Denmark; 5 OPEN - Open Patient data Explorative Network, Odense University Hospital, Odense, Denmark; 6 Department of Endocrinology, Odense University Hospital, Odense, Denmark

**Keywords:** Diabetes in pregnancy, Gestational diabetes, Register-based cohort study, Breast cancer, Premenopausal breast cancer, Postmenopausal breast cancer

## Abstract

**Background:**

Gestational diabetes mellitus (GDM) is a common pregnancy complication characterized by insulin resistance. A link has been suggested between insulin resistance and breast cancer, which is the most common cancer in women. Hence, women with previous GDM may be at increased risk of developing breast cancer, yet, the existing evidence is conflicting. This study explored the association between GDM and incident breast cancer, including age at cancer diagnosis. Additionally, we investigated the potential impact of severity of insulin resistance during pregnancy and of subsequent diabetes development on the breast cancer risk.

**Methods:**

We conducted a nationwide, register-based cohort study including all women giving birth in Denmark from 1997 to 2018. We defined GDM and breast cancer based on ICD-10 codes. Premenopausal and postmenopausal breast cancer was pragmatically defined as age at outcome < 50 years and ≥ 50 years, respectively. A proxy for severity of insulin resistance during pregnancy was based on insulin treatment; subsequent diabetes was defined as presence of ICD-10 codes and/or antidiabetic medication after pregnancy. The statistical analyses included Cox regression, logistic regression and t-test.

**Results:**

Of 708,121 women, 3.4% had GDM. The median follow-up period was 11.9 years (range 0-21.9). The overall breast cancer risk was comparable in women with and without previous GDM (adjusted hazard ratio 0.96 [95% CI 0.83–1.12]). Premenopausal and postmenopausal breast cancer risk also did not differ; however, women with previous GDM had a breast cancer diagnosis at younger age (42.6 vs. 43.5 years, *p*-value 0.01). All-cause mortality was similar regardless of GDM history. Severity of insulin resistance during pregnancy and subsequent diabetes did not affect breast cancer risk.

**Conclusions:**

This large, population-based cohort study showed no higher risk of incident breast cancer in women with previous GDM compared to women without previous GDM after a median of almost 12 years of follow-up. This was evident irrespective of menopausal state. The breast cancer risk was not influenced by the severity of insulin resistance during pregnancy and by subsequent diabetes development. Regardless of GDM history, attention towards prevention, early detection and treatment of breast cancer should be prioritized.

**Supplementary Information:**

The online version contains supplementary material available at 10.1186/s13058-024-01908-4.

## Background

Gestational diabetes mellitus (GDM) complicates millions of pregnancies worldwide every year with an estimated global prevalence of 14% based on large variations internationally, yet with rising prevalence across the world [1]. GDM is a condition representing varying levels of insulin resistance and hyperglycemia. Physiologically, insulin resistance increases during pregnancy, however, GDM and maternal hyperglycemia arise in case of an insufficient insulin response combined with a relative beta-cell dysfunction [2]. Normoglycemia is targeted through changes in diet and lifestyle initially, but in case of profound insulin resistance, pharmacological medication (primarily insulin) is recommended as part of GDM treatment [2].

Maternal and neonatal complications during pregnancy and labor are more frequent among women with GDM compared to women with normoglycemia [2]. GDM generally disappears after delivery, however, even many years beyond pregnancy, GDM associates with a wide range of impaired health conditions; e.g., a ten-fold risk of diabetes (mainly type 2 diabetes) [3], a two-fold risk of cardiovascular disease [4] and of chronic kidney disease [5], and a 1.2-fold risk of psychiatric morbidity [6]. The mechanism between GDM and future morbidity is likely multifactorial, and insulin resistance has been suggested as a common factor underlying associations between past and future compromised health [2]. This proposal is supported by evidence that women with increasing severity of insulin resistance during pregnancy (defined by insulin treatment during GDM pregnancy) have higher morbidity risk later in life; even in the absence of subsequent diabetes [5, 6].

Insulin resistance has been linked to development of cancer in general [7] and to breast cancer specifically [8]. The association potentially arises through a complex interplay between metabolic dysregulation and cancer pathogenesis, where insulin resistance induces a compensatory hyperinsulinemia, which may promote cancer cell proliferation, cancer development, and progression [7–9]. An umbrella review found evidence of an association between type 2 diabetes and breast cancer [10]. However, findings from a Mendelian randomization study indicated that such association may be ascribed to insulin resistance rather than type 2 diabetes per se [11]. A review exploring the relation between obesity, menopausal state, and breast cancer concluded that paradoxically, obesity associates with lower risk of premenopausal breast cancer and higher risk of postmenopausal breast cancer [12]. As obesity is a significant risk factor for GDM [1], women with previous GDM may similarly face a lower risk of premenopausal breast cancer and a higher risk of postmenopausal breast cancer. Breast cancer constitutes a prevalent malignancy and contributes significantly to female mortality [13]. It is therefore crucial to elucidate the risk of breast cancer in women exposed to the increasingly common, insulin resistant state of GDM, as the existing evidence provides conflicting conclusions [14–16].

Thus, the overall aim was to explore breast cancer risk according to previous GDM in a nationwide population of women giving birth in Denmark from 1997 to 2018. The objectives were to investigate: (1) whether women with GDM compared to women without GDM had higher risk of incident breast cancer (overall, premenopausal and postmenopausal breast cancer) and earlier age at diagnosis; (2) the potential impact of severity of insulin resistance during pregnancy on the breast cancer risk; and (3) the potential impact of subsequent diabetes development on the association between GDM and breast cancer.

## Methods

### Study design and data sources

We conducted a nationwide, register-based cohort study with data sources consisting of prospectively collected data from the national Danish registers containing data on the complete population of Danish residents [17–22]. In Denmark, each individual is assigned a personal identification number hereby facilitating individual-level record linkage of data from the registers [17]. We used the Danish Medical Birth Register [18] for identifying the study population and for data regarding pregnancy and delivery. The Danish National Patient Registry [19] contains data on all hospital contacts and contributed with data on ICD-10 diagnosis codes. Data on redemptions of prescribed medication derived from the Danish National Prescription Registry [20], which holds information on all prescriptions redeemed at community pharmacies by Danish residents. We collected demographic and socioeconomic data from the following registers: the Danish Civil Registration System [17], the Income Statistics Register [21], and the Population Education Register [22].

### Study population

**Fig. 1:**
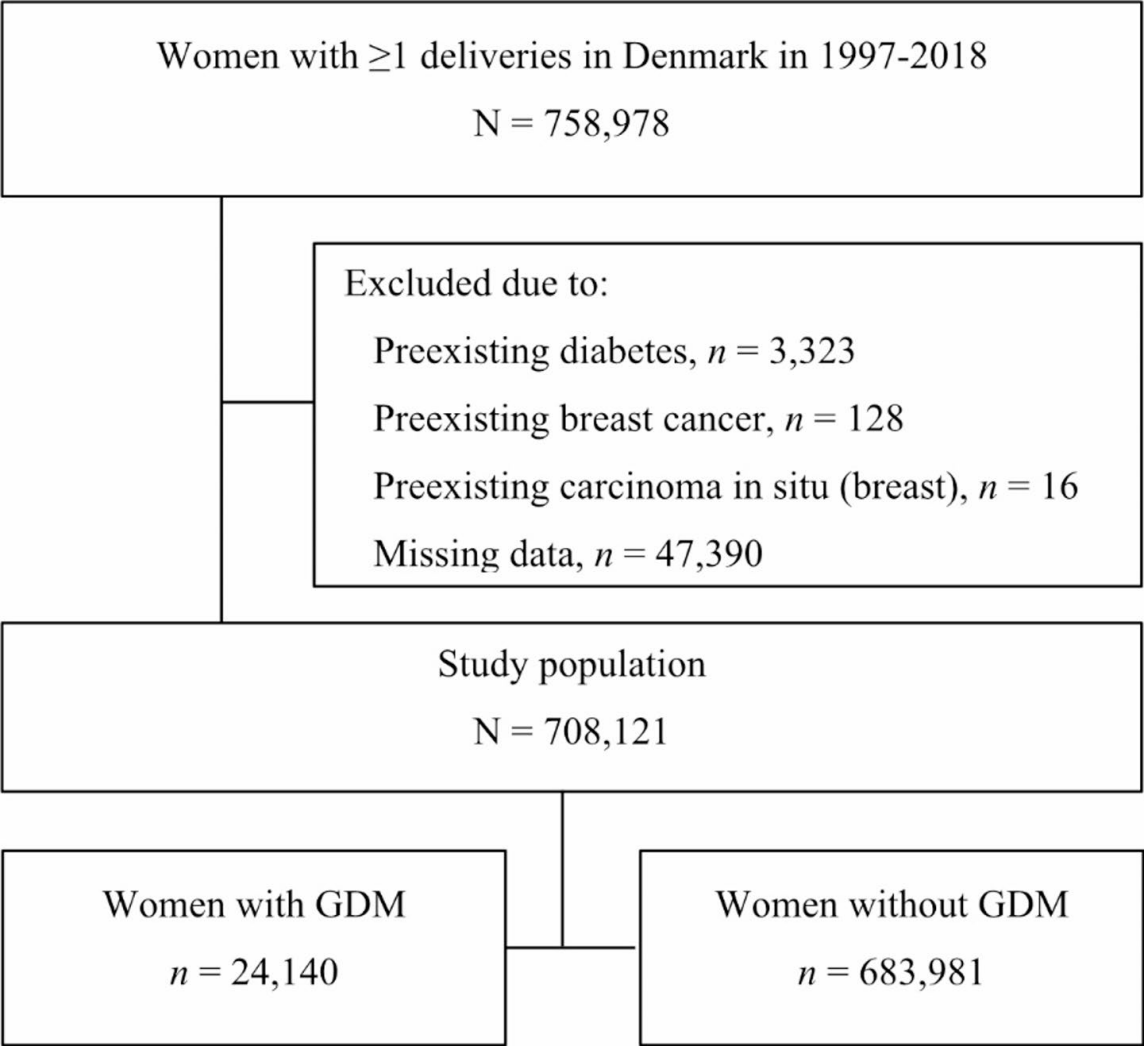
Flowchart of the study population. Abbreviation: GDM, gestational diabetes mellitus

All Danish residents giving birth in Denmark during the study period from 1/1-1997 to 31/12-2018 were eligible for inclusion in the study population. We excluded women with preexisting diabetes based on diagnosis codes and/or redemption of prescribed antidiabetic agents (except for metformin). Women treated with metformin prior to pregnancy without a diabetes diagnosis were perceived as having polycystic ovary syndrome (PCOS) and were not excluded as PCOS is a risk factor for GDM [2]; however, clinical practice in Denmark involves pausation of metformin treatment during pregnancy and breastfeeding. Likewise, we excluded women who already had a diagnosis of breast cancer or carcinoma in situ in the breast. The exclusion criteria window was the time period from two years prior to the index date and until the index date (defined as the conception date in the index pregnancy, i.e., the first pregnancy during the study period). This choice arose as data were eligible from 1995 (i.e., two years prior to commencement of the study period) and as we required identical exclusion criteria window regardless of time of study entry. Finally, we excluded women with missing data regarding the covariates that a priori were decided to be included in the statistical analyses. Women remained in the study population if they experienced fetal loss during pregnancy (i.e., after gestational week 28 prior to 2004 and after gestational week 22 from 2004 onwards due to a change in registration practice [18]) or fetal loss during delivery. Figure 1 illustrates the study population flowchart and Supplementary Table 1 lists all definitions and categorizations.

### GDM exposure

GDM exposure was based on ICD-10 code O24.4. In Denmark, GDM screening is selective based on risk factors (BMI ≥ 27 kg/m^2^, glucosuria, family history of diabetes, previous GDM, previous delivery of newborn ≥ 4500 g, PCOS or current multiple pregnancy). The diagnostic test is a 2-hour 75 g oral glucose tolerance test (OGTT) in gestational weeks 24–28 [23]. An additional OGTT is recommended in gestational weeks 10–20 in case of previous GDM and/or ≥ 2 risk factors. The diagnostic criteria is a 2-hour glucose value ≥ 9.0 mmol/L (in venous plasma or capillary blood) [23]. As the women in the study population contributed with ≥ 1 pregnancies during the study period, we handled GDM as a time-varying exposure in the survival analysis thereby allowing for potential change of exposure status across pregnancies.

### Breast cancer outcome

Breast cancer outcome was defined by ICD-10 code C50. Furthermore, we defined premenopausal and postmenopausal breast cancer by age at diagnosis < 50 years and ≥ 50 years, respectively. In Denmark, all women aged 50–69 years are offered mammography for breast cancer screening every second year and the attendance rate is high (84% in 2018–2020) [24].

### Follow-up and risk time

Follow-up began six weeks after the delivery date in index pregnancy resembling the end of the postpartum period. Risk time continued from this date and until outcome, emigration, death, or end of study period, whichever came first. The contribution of risk time paused from conception date to six weeks postpartum during any subsequent pregnancies. We categorized the risk time as exposed or unexposed depending on GDM exposure. However, once GDM occurred, all subsequent risk time was considered as exposed.

### Covariates

We defined potential confounders a priori based on existing literature (Supplementary Table 1). The variables were clinical, demographic, and socioeconomic factors derived from the index pregnancy: maternal age, parity, preexisting hypertension, preexisting comorbidity based on the Charlson Comorbidity Index [25], ethnicity, marital status, income, occupation, education, and calendar year of delivery. Adjustments for pregestational BMI and smoking during pregnancy were reserved for sensitivity analyses as these registrations did not commence until late 2003 and late 1997, hence resulting in missing data.

For exploring the potential impact of increasing severity of insulin resistance during pregnancy, we generated a proxy variable based on GDM and insulin treatment during pregnancy hereby constructing a variable composed of three categories representing increasing insulin resistance: (1) no GDM; (2) GDM and no insulin treatment; and (3) GDM and insulin treatment. Concomitantly, we investigated the impact of insulin resistance during pregnancy in women with and without subsequent diabetes development. Subsequent diabetes was defined as incident diabetes after pregnancy and prior to breast cancer outcome and was based on diagnosis codes and/or redemption of antidiabetic medication (Supplementary Table 1).

### Statistical analyses

We compared baseline characteristics using the Wilcoxon Rank Sum test and the Chi-squared test for continuous and categorical variables, respectively. Cox regression models were performed to investigate the association between GDM and breast cancer by estimating crude hazard ratios (HR) and adjusted hazard ratios (aHRs) with 95% confidence intervals (CIs). Clustering on each woman allowed us to account for potential repeated measurements related to contribution of more than one pregnancy by each woman during the study period. We tested the assumptions for Cox regression analysis by the Schoenfeld residuals. Non-proportionality was handled if required by including interaction terms between the covariates and the time period in the final adjusted model. We included the proxy variable for severity of insulin resistance during pregnancy as interaction term with subsequent diabetes in the adjusted Cox regression model in order to explore their potential impact on breast cancer risk. We performed two-sample t-tests and logistic regression analyses for comparison of age at breast cancer diagnosis and all-cause mortality in women with incident breast cancer according to GDM history.

Missing data were handled via the exclusion criteria regarding the a priori selected confounders; via sensitivity analyses regarding pregestational BMI and smoking during pregnancy (see below); or via imputation of the mean value regarding gestational age at delivery (< 2%).

We performed several sensitivity analyses. First, we expanded the confounder adjustment by including additional potential confounders from index pregnancy in separate analyses: Pregestational BMI, smoking during pregnancy, preeclampsia and/or gestational hypertension, preterm delivery, and preexisting metformin treatment. Further, we excluded women with any preexisting cancer and/or any preexisting carcinoma in situ. Finally, we included time spent during pregnancy and the immediate postpartum periods as risk time in the follow-up.

Stata 18 software (StataCorp LLC, College Station, TX, USA) was used for statistical analyses. *P*-values < 0.05 were considered statistically significant.

## Results

### Study population

During the study period, 759,978 women gave birth. After exclusions, the study population consisted of 708,121 women of whom 24,140 (3.4%) were diagnosed with GDM in ≥ 1 pregnancy (Fig. 1). Insulin treatment occurred in 3,114 women with GDM (12.9%). We based the Cox regression analyses on data on 704,608 women as we omitted women diagnosed with breast cancer from index/conception date to six weeks postpartum. The median follow-up period was 11.9 years (range 0–21.9 years); total risk time was 8,135,323 years. Subsequent diabetes developed in 4,812 (20.1%) and 13,600 (2.0%) women with and without previous GDM, respectively.

### Baseline data

Women with GDM differed significantly from women without GDM with regard to several characteristics in index pregnancy (Table 1); they had higher pregestational BMI (27.2 kg/m^2^ vs. 22.8 kg/m^2^) and were more likely to have preexisting morbidities (e.g., hypertension [2.5% vs. 1.3%], and metformin treatment [2.8% vs. 0.7%]), however, carcinoma in situ was less prevalent (0.1% vs. 0.2%). Socioeconomic factors also differed significantly, e.g., women with GDM were more often of non-Danish ethnicity (21.6% vs. 11.9%), had the lowest educational level (24.4% vs. 19.3%), and were less likely to be in the active workforce (68.3% vs. 71.9%). However, there were no significant differences regarding smoking and preexisting cancer. Finally, obstetric complications occurred more often in women with GDM.

**Table 1:** Baseline characteristics from index pregnancy according to GDM history

	GDM (*n* = 24,140)	No GDM (*n* = 683,981)	*P* value
**Clinical characteristics**			
Age (years)	28 (25**–**32)	28 (25**–**31)	< 0.001
Primiparity	20,277 (84.0)	531,526 (77.7)	< 0.001
Pregestational BMI (kg/m^2^)^a^	27.2 (23.1**–**31.6)	22.8 (20.7**–**25.8)	< 0.001
Smoking during pregnancy^b^	3,832 (17.4)	102,931 (17.4)	0.841
Preexisting hypertension	594 (2.5)	8,746 (1.3)	< 0.001
Preexisting metformin treatment	674 (2.8)	5,015 (0.7)	< 0.001
Preexisting cancer (not breast)	38 (0.2)	1,142 (0.2)	0.721
Preexisting carcinoma in situ (not breast)	31 (0.1)	1,310 (0.2)	0.027
No preexisting comorbidity^c^	23,798 (98.6)	677,242 (99.0)	< 0.001
**Demographic characteristics**			
Ethnicity			
Danish	19,169 (79.4)	602,906 (88.1)	< 0.001
Immigrant, Western	625 (2.6)	21,473 (3.1)	< 0.001
Immigrant, Non-Western	3,745 (15.5)	49,138 (7.2)	< 0.001
Descendant^d^	601 (2.5)	10,464 (1.5)	< 0.001
Marital status			
Single/not living with a partner	2,914 (12.1)	84,200 (12.3)	0.266
Married/living with a partner	21,226 (87.9)	599,781 (87.7)	0.266
Income, tertile			
Low	7,968 (33.0)	214,526 (31.4)	< 0.001
Middle	7,694 (31.9)	234,543 (34.3)	< 0.001
High	8,478 (35.1)	234,912 (34.3)	0.013
Highest completed education			
Lower secondary	5,891 (24.4)	132,315 (19.3)	< 0.001
Upper secondary	10,064 (41.7)	287,527 (42.0)	0.283
Post secondary	8,185 (33.9)	264,139 (38.6)	< 0.001
Occupation			
Employed	16,495 (68.3)	491,945 (71.9)	< 0.001
Unemployed or on welfare payment	2,759 (11.4)	89,650 (13.1)	< 0.001
Under education	3,160 (13.1)	68,731 (10.0)	< 0.001
Early retirement	274 (1.1)	3,203 (0.5)	< 0.001
**Obstetrical characteristics**			
Preeclampsia	1704 (7.1)	23,850 (3.5)	< 0.001
Gestational hypertension	966 (4.0)	11,019 (1.6)	< 0.001
Preterm delivery	2473 (10.2)	46,119 (6.7)	< 0.001

Abbreviations: GDM, gestational diabetes mellitus; BMI, body mass index

Data presented as median (interquartile range) or number (%)

^a^*n* = 375,932

^b^*n* = 610,932

^c^ Charlson Comorbidity Index score of 0

^d^ Person born in Denmark by parents who were born outside of Denmark and without Danish citizenships

### Incidence of breast cancer

Table 2 shows results regarding incidence of breast cancer. Compared to women without GDM, the aHRs for breast cancer in women with GDM was 0.96 (95% CI 0.83–1.12). For premenopausal and postmenopausal breast cancer, the risk estimates were likewise statistically insignificant. The sensitivity analyses did not produce substantially different risk estimates.

Women with incident breast cancer were diagnosed at lower age in case of previous GDM compared to no previous GDM (42.6 years vs. 43.5 years, respectively; *p* *=* 0.01). All-cause mortality after breast cancer diagnosis was not related to GDM history (adjusted odds ratio 1.28 [95% CI 0.81–2.04]).

**Table 2:** Risk of breast cancer according to GDM history

	GDM		No GDM		HR (95% CI)	
	Events, *n*	Incidence rate^a^ (95% CI)	Events, *n*	Incidence rate^a^ (95% CI)	Crude	Adjusted^b^
**Breast cancer**	174	0.9 (0.8–1.1)	7,435	0.9 (0.9-1.0)	0.99 (0.85–1.15)	0.96 (0.83–1.12)
Premenopausal breast cancer^c^	145	0.8 (0.7–0.9)	5,955	0.7 (0.7–0.8)	1.01 (0.86–1.19)	0.99 (0.84.1.17)
Postmenopausal breast cancer^d^	29	0.2 (0.1–0.2)	1,480	0.2 (0.2–0.2)	0.90 (0.62–1.30)	0.86 (0.59–1.24)

Abbreviations: GDM, gestational diabetes mellitus; HR, hazard ratio

^a^ Incidence rate presented as number of events per 1,000 person-years

^b^Adjusted for age at index pregnancy, parity, preexisting hypertension, preexisting comorbidity, ethnicity, marital status, income, education, occupation, and calendar year of delivery

^c^Age at diagnosis < 50 years

^d^Age at diagnosis ≥ 50 years

### Severity of insulin resistance and breast cancer risk

**Fig. 2:**
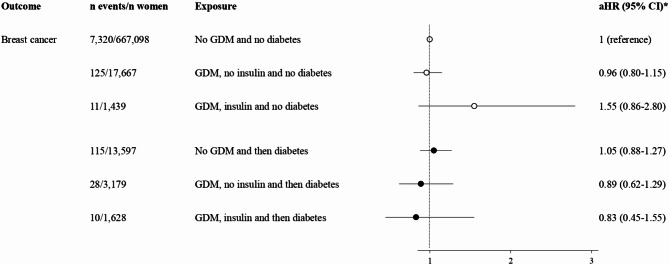
Breast cancer risk according to GDM, severity of insulin resistance, and subsequent diabetes. Abbreviations: GDM, gestational diabetes mellitus; aHR, adjusted hazard ratio. White circles = women without subsequent diabetes. Black circles = women with subsequent diabetes. *Adjusted for age at index pregnancy, parity, preexisting hypertension, preexisting comorbidity, ethnicity, marital status, income, education, occupation, and calendar year of delivery

Figure 2 illustrates that the breast cancer risk in women with non-insulin-treated and insulin-treated GDM equaled the risk in the reference group of women without GDM; the aHRs reached 0.96 (95% CI 0.80–1.15) and 1.55 (95% CI 0.86–2.30) respectively, when analyzing data in women without subsequent diabetes. The same insignificant pattern was evident in the presence of subsequent diabetes. Regarding premenopausal and postmenopausal breast cancer, all risk estimates were insignificant and displayed similar pattern to the overall breast cancer risk (data not shown).

### Subsequent diabetes and breast cancer risk

The results in Table 3 show that the risk of breast cancer (overall, premenopausal, and postmenopausal) did not differ significantly in relation to subsequent diabetes development, regardless of previous GDM. For breast cancer in the presence of subsequent diabetes, the aHRs were 0.88 (95% CI 0.64–1.21) and 1.05 (95% CI 0.88–1.27) in women with and without previous GDM, respectively. Accordingly, all risk estimates were insignificant regarding premenopausal and postmenopausal breast cancer.

**Table 3:** Risk of breast cancer according to GDM history and subsequent diabetes development

	Crude HR (95% CI)	Adjusted HR (95% CI)^a^
**Breast cancer**		
No GDM and no diabetes	1 (reference)	1 (reference)
GDM and no diabetes	1.03 (0.87–1.22)	0.99 (0.83–1.17)
No GDM and then diabetes	1.03 (0.86–1.24)	1.05 (0.88–1.27)
GDM and then diabetes	0.89 (0.64–1.22)	0.88 (0.64–1.21)
**Premenopausal breast cancer** ^**b**^		
No GDM and no diabetes	1 (reference)	1 (reference)
GDM and no diabetes	1.05 (0.87–1.26)	1.01 (0.84–1.22)
No GDM and then diabetes	0.93 (0.74–1.17)	0.97 (0.77–1.22)
GDM and then diabetes	0.89 (0.62–1.28)	0.91 (0.63–1.31)
**Postmenopausal breast cancer** ^**c**^		
No GDM and no diabetes	1 (reference)	1 (reference)
GDM and no diabetes	0.93 (0.60–1.44)	0.92 (0.59–1.43)
No GDM and then diabetes	1.29 (0.94–1.76)	1.10 (0.80–1.52)
GDM and then diabetes	0.87 (0.45–1.68)	0.75 (0.39–1.46)

Abbreviations: GDM, gestational diabetes mellitus; HR, hazard ratio

^a^ Adjusted for age at index pregnancy, parity, preexisting hypertension, preexisting comorbidity, ethnicity, marital status, income, education, occupation, and calendar year of delivery

^b^ Age at diagnosis < 50 years

^c^ Age at diagnosis ≥ 50 years

## Discussion

This nationwide cohort study including over 700,000 parous women with a median follow-up period of almost 12 years showed that women with GDM had no higher risk of developing breast cancer, neither overall breast cancer, nor premenopausal or postmenopausal breast cancer when compared to women without previous GDM. However, women with breast cancer were younger at diagnosis in case of previous GDM. Severity of insulin resistance during pregnancy and subsequent diabetes development did not influence the breast cancer risk.

Our finding of no association between GDM and breast cancer is in line with systematic reviews and meta-analyses [14–16] indicating that there is no firm evidence of an overall association. The studies included in the systematic reviews and meta-analyses contributed with conflicting results showing either lower, higher or unaffected breast cancer risk according to GDM history based on different study designs, study population sizes, ethnicities, GDM prevalence and follow-up durations [14–16]. One of the systematic reviews [15] reported that study design seemed to have an impact as no association was found in case-control studies versus a moderately higher risk in cohort studies (odds ratio 1.25 [95% CI 1.00-1.56]). Another systematic review/meta-analysis [16] reported no impact of study design but rather of region with only women from Asia being at risk (relative risk 1.31 [95% CI 1.01–1.70]). Correspondingly, our finding of no association may not surprise, as our study was a cohort study including primarily Caucasian women. In contrast, cohort studies from the U.S [26]. and Canada [27] reported a lower risk after GDM, whereas a cohort study from Israel [28] found a higher breast cancer risk. Explanations for these discrepant findings are elusive as they derive from studies of good quality with validated data sources [26–28]. Different strategies regarding confounder adjustment may to some extent influence the diverging results as the studies finding lower risk [26, 27] were able to conduct a more extensive adjustment strategy compared to the study finding higher risk [28] and also compared to our study. Likewise, differences in GDM screening strategies, GDM diagnostic criteria, demographic characteristics and overall health care services may influence the results. Interestingly regarding impact of regional differences, cohort studies from South Korea (i.e., representing an Asian region) have despite relatively similar follow-up durations reported same risk [29] and higher risk [30] after GDM with and without accounting for confounders, respectively. Hence, when investigating the relationship between GDM and breast cancer, it seems of paramount importance to be able to provide a highly nuanced confounder adjustment strategy.

Regarding premenopausal breast cancer, our study is the largest population-based cohort study with up to 22 years of follow-up addressing the risk of incident premenopausal breast cancer after GDM and contributes with the robust finding of no association, even after accounting for pregestational BMI. Contrastingly, other studies have found a significantly lower risk of 32% and 14% [26, 27]. A cohort study from the U.S. including ∼ 87,000 women [26] found a decreased risk regardless of menopausal state whereas a Canadian study with ∼ 150,000 women only included women aged 20–50 years [27] resembling premenopausal women. The reason for the discrepancies in findings across studies is likely multifactorial and reflects the high complexity that characterizes research in associations between GDM, diabetes, obesity, and breast cancer with the vast amount of shared pathways as well as confounding and modifying factors. However importantly, our study contributed with the message that GDM was not related to a lower risk of premenopausal breast cancer, which therefore warrants similar attention irrespective of GDM history.

Regarding postmenopausal women, a study on an Israeli cohort with ∼ 38,000 women (1% with GDM) and a median follow-up of 34 years found a 70% higher breast cancer risk after GDM in women above 50 years [31], In contrast, the previously mentioned U.S.-cohort study found a 37% lower risk in postmenopausal women after 22 years follow-up [26] and similarly, a case-control study from the U.S. including ∼ 2,400 women (3–4% self-reporting GDM) found a 40% lower risk in postmenopausal women [32]. Hence, the existing evidence is conflicting and may be explained by differences in age/follow-up period since menopause and age are closely related and age is a crucial risk factor for breast cancer [33]. Our finding of no significant link between GDM and postmenopausal breast cancer might be influenced by the relatively young median age of our study population (50 years) at the end of follow-up, despite the long follow-up period. In our study population, a statistically significant association might appear if follow-up was extended so that a large proportion of the women reached the age of, e.g., 70 years or more, hereby providing more statistical power for exploration of such association; especially if current BMI/obesity level could be taken into account [12].

The finding of nearly a one-year younger age at breast cancer diagnosis in women with incident breast cancer after GDM is intriguing and warrants further investigation. Our study did not determine the underlying reasons for this difference. It could be hypothesized that it is influenced by differences in preexisting clinical risk factors that may pose additional challenges to the future health in women with previous GDM in whom the metabolic dysfunction to some extent may persist beyond pregnancy [2] and be involved in hormonal imbalances. Also, differences in chronic inflammatory responses and immune responses may have impact [7]. As such, it remains unclear whether the observed difference reflects earlier cancer development per se, earlier detection of the condition due to increased surveillance or merely a chance finding. Additionally, it is unknown whether the difference in age at diagnosis affects breast cancer treatment, progression and recovery in the afflicted women. However, it is reassuring that women with breast cancer and a history of GDM did not have significantly higher all-cause mortality during the study period compared to women with breast cancer and no previous GDM. Of notice, our findings are restricted to the age reached by the women in the study population during the investigated time period and as age is an important risk factor for breast cancer [33], the complete relationship between GDM and age at breast cancer diagnosis is potentially not accounted for by our study.

This study contributes with novel knowledge indicating that the severity of insulin resistance during pregnancy was not related to the future breast cancer risk. The lack of dose-response relationship conflicted with our initial hypothesis based on the association between insulin resistance and breast cancer [8]. GDM is normally a condition of relatively modest and temporary insulin resistance with glucose metabolism restoring postpartum [2] and it could be speculated that the insulin resistance constituted by GDM per se did not impose substantial, cumulative impact on the breast cancer risk as seen in women with manifest diabetes and chronic insulin resistance [10, 11] – regardless of our subcategories of severity of insulin resistance during pregnancy. In keeping with our finding, a meta-analysis concluded that prediabetes (representing another insulin resistant condition of confined severity) did not associate with the risk of breast cancer [34].

We additionally explored the role of subsequent diabetes development in relation to previous GDM and our data did not provide evidence for a higher breast cancer risk in women with subsequent diabetes regardless of GDM history. This contrasts with the conclusion from the umbrella review on type 2 diabetes and cancer [10], but is in correspondance with findings from the Mendelian randomization study [11]. In our study, we did not differentiate into subtypes of subsequent diabetes; however, the majority of incident diabetes in our study population expectedly comprised type 2 diabetes [3]. Pathophysiologically, sub-phenotypes of type 2 diabetes have been identified and demonstrate that type 2 diabetes is characterized by varying levels of insulin resistance and beta-cell function, hereby representing vast heterogeneity with regard to hypo-/hyperinsulinemia, but also with regard to obesity [35]. Insulin is a potential oncogenic factor involved in stimulation of cell proliferation, including malignant tumor cells [7]. Hence, specific subgroups of type 2 diabetes may bear different risks of cancer development due to underlying heterogeneity of hypo-/hyperinsulinemia and/or insulin resistance, potentially influenced by obesity level. However, insulin treatment per se in women with diabetes does not seem to be related to breast cancer risk [36]. Discrepancies in findings across studies regarding the association between diabetes and breast cancer could potentially be partly explained by different distributions of sub-phenotypes of type 2 diabetes and adiposity, yet this needs further exploration.

Strengths of our study include the large study population of more than 700,000 women and the long follow-up duration of up to 22 years. The population-based approach in combination with the universal health care system providing tax-supported health care for the entire Danish population entailed a minimization of selection bias. Further, the register data in the Danish registers are prospectively collected and considered a valid data source for epidemiological research [18–20] with high validity of the GDM diagnosis code [37] as well as of cancer diagnosis codes, including breast cancer [38]. Using the individual woman as study unit and including all pregnancies by each woman during the complete study period, we were able to create a nuanced GDM exposure assessment over time, as it was not restricted to a random pregnancy. Additionally, we accounted for relevant confounders.

The study has certain limitations. Importantly, the GDM prevalence may be underestimated due to the selective GDM screening strategy and women might be misclassified as non-GDM, which potentially generated an underestimated breast cancer risk according to GDM exposure. Furthermore, GDM may constitute an undiagnosed pregestational diabetes and hence be misclassified; the implication of this is unknown. The long follow-up period implies potential changes in screening strategies regarding both GDM and breast cancer. Concerning GDM, diminutive changes were made in 2003 [23], however, our findings are expectedly not impacted hereby. The breast cancer screening strategy was implemented nationally in 2009 but was introduced in 1991 and performed in large parts of the country from 1994 onwards. We assumed that breast cancer screening attendance is unrelated to GDM history and hence do not anticipate impact on the conclusions. Detection bias may be present as women with GDM were recommended to attend follow-up at their general practitioner with 1–3 year intervals after delivery. However, as the follow-up attendance rate is relatively low [39] and as breast cancer screening is offered to all women for free from the age of 50 years and has a very high attendance rate [24], the potential consequence of detection bias is expectedly negligible. Due to the study design, we were not able to distinguish between different subtypes of breast cancer or the timing in relation to menopausal state beyond the pragmatic strategy of considering 50 years of age as cut-off for premenopausal versus postmenopausal breast cancer. We also generated a proxy for severity of insulin resistance during pregnancy based on insulin treatment, yet arguably, the proxy may rather represent beta-cell dysfunction or a combination of the two. However, perceiving normal pregnancy as a relatively insulin resistant state, the concept of insulin resistance remained our focus and furthermore, the available register data did not allow for further elucidation of the heterogeneous condition. Contemplating our clinical experience, the proxy was challenged by unexpected low prevalence of insulin treatment in our data. The implication may be an underestimated impact of increasing insulin resistance. Finally, the confounder data originated from the index pregnancy and some were self-reported. Consequently, some may not characterize the woman later in life and/or may be misreported. Lastly, we did not have access to information regarding physical activity, family history of cancer and diabetes, breastfeeding history, BMI trajectory, age at menarche and menopause, contraceptive and postmenopausal medication, and other relevant factors that potentially confound the results. The generalizability of the study findings is potentially limited to settings characterized by similar ethnic composition (with primarily Caucasians), health care system (with provision of many health care services for free), and breast cancer screening (with automated invitations).

## Conclusions

To conclude, this large population-based study from Denmark with a median follow-up period of almost 12 years showed no association between GDM and incident breast cancer, neither overall breast cancer, nor premenopausal or postmenopausal breast cancer. However, the potential association between GDM and postmenopausal breast cancer needs further exploration in large studies with longer follow-up period than our study could provide, ideally taking the impact of BMI/obesity into account as well as other relevant confounders. Additionally, the observed difference in age at breast cancer diagnosis in relation to previous GDM warrants further investigation. The clinical implication of our study is that regardless of GDM history, breast cancer awareness and early detection should be prioritized.

## Electronic supplementary material

Below is the link to the electronic supplementary material.

Supplementary Material 1

## Data Availability

No datasets were generated or analysed during the current study.

## Abbreviations

aHR: Adjusted hazard ratio
ATC: Anatomical Therapeutic Chemical groups
BMI: Body mass index
CCI: Charlson comorbidity index
CI: Confidence interval
GDM: Gestational diabetes mellitus
HR: Hazard ratio
ICD-10: International classification of diseases, 10th revision
IQR: Interquartile range
PCOS: Polycystic ovary syndrome
