# Supplementary material for: Breast cancer in women with previous gestational diabetes: a nationwide register-based cohort study
Source: Breast Cancer Res. 2024 Nov 4;26:150. doi: 10.1186/s13058-024-01908-4 (PMC11533352; doi:10.1186/s13058-024-01908-4)
Supplement: Supplementary file 1 — Supplementary Material 1 [file 13058_2024_1908_MOESM1_ESM.docx]

**Supplementary Table 1** – Definitions and categorizations of variables according to the ICD-10 codes (International Classification of Diseases, 10^th^ revision) and the ATC groups (Anatomical Therapeutic Chemical groups)

|  | **ICD-10 codes and ATC groups** |
| --- | --- |
| **Exclusion criteria** | **At or 2 years before index date** |
| Preexisting diabetes | ICD-10: E10-E14, O240-O243, O245, O249 *and/or*  ATC: A10 (except A10BA02) (≥2 redemptions) |
| Preexisting breast cancer:  Preexisting carcinoma in situ in the breast | ICD-10: C50  ICD-10: D05 |
| Additional exclusion criteria for sensitivity analysis:  Any preexisting cancer  Any preexisting carcinoma in situ | ICD-10: C00-C99  ICD-10: D00-D09 |
| **Exposure** |  |
| Gestational diabetes mellitus (GDM) | ICD-10: O244 |
| **Outcomes** | **From 6 weeks postpartum** |
| Breast cancer | ICD-10: C50 |
| **Covariates** | **Data from index pregnancy unless stated otherwise** |
| Maternal age | Age in years at date of delivery |
| Primiparity  Parity | No deliveries prior to index pregnancy  For confounder adjustment, parity was entered as a time-varying confounder in the Cox regression models |
| Pregestational BMI | Selfreported data or measured at first antenatal visit |
| Smoking during pregnancy | Selfreported data |
| Preexisting hypertension | ICD-10: I10-11, I152-9 (within 2 years prior to index date until gestational week 20) *and/or*  ATC: C02-C03, C07-C09 (≥2 redemptions within 2 years prior to index date until gestational week 20) |
| Preexisting metformin treatment | ATC: A10BA02 (≥2 redemptions within 2 years prior to index date) |
| Preexisting cancer (not breast) | C00-C49, C51-C99 |
| Preexisting carcinoma in situ (not breast) | D00-D04, D06-D09 |
| No preexisting comorbidity | Charlson Comorbidity Index score of 0 |
| Ethnicity  *Danish*  *Immigrant, Western*  *Immigrant, Non-Western*  *Descendant* | Combination of data on ethnicity/immigration status and country of origin and divided into four categories:  Born in Denmark or abroad by parents where at least one parent is born in Denmark and holds Danish citizenship  Born in ‘Other Western countries’ by parents born outside of Denmark and without Danish citizenship  Born in ‘Non-Western countries’ by parents born outside of Denmark and without Danish citizenship  Born in Denmark by parents that were born outside of Denmark and who do not hold Danish citizenships |
| Marital status | Combination of data on marital status and family type and divided into two categories (Single/not living with a partner, Married/living with a partner) |
| Income | In the calendar year prior to delivery based on a format by Statistics Denmark and divided into three categories (Low, Middle, High) |
| Highest completed education | Based on a format by Statistics Denmark and divided into three categories (Lower secondary, Upper secondary, Post secondary) |
| Occupation | Based on data on connection to the workforce by Statistics Denmark and divided into four categories (Employed, Unemployed or on welfare payment, Under education, Early retirement) |
| Preeclampsia | ICD-10: O11, O14 (from gestational week 20 to 6 weeks postpartum) |
| Gestational hypertension | ICD-10: O13, O16 (from gestational week 20 to 6 weeks postpartum) |
| Preterm delivery | Delivery prior to gestational week 37 |
| **Other variables** |  |
| Insulin treatment (in any GDM pregnancy) | ICD-10: O244E *and/or*  ATC: A10A (≥1 redemption in ≥1 GDM pregnancy) |
| *Proxy for level of insulin resistance*  1. No GDM  2. GDM and no insulin treatment  3. GDM and insulin treatment | *Increasing severity with category 3 being the most severe:*  1. No GDM diagnosis in any pregnancy  2. GDM in ≥1 pregnancy, no insulin treatment (see above)  3. GDM in ≥1 pregnancy and insulin treatment (see above) |
| Subsequent diabetes after index pregnancy and prior to breast cancer | ICD-10: E10-E14, O240-O243, O245, O249 *and/or*  ATC: A10 (≥2 redemptions) *and*  no incident breast cancer prior to subsequent diabetes |
